# Factors enhancing the level of utilisation of research knowledge on ecosystems

**DOI:** 10.1371/journal.pone.0254752

**Published:** 2021-07-22

**Authors:** René Eschen, Purity Rima Mbaabu, Bruno Salomon Ramamonjisoa, Carmenza Robledo-Abad

**Affiliations:** 1 CABI, Delémont, Switzerland; 2 Kenya Forestry Research Institute (KEFRI), Marigat, Kenya; 3 Institute for Climate Change and Adaptation (ICCA), University of Nairobi, Nairobi, Kenya; 4 Faculty of Humanities and Social Sciences, Chuka University, Chuka, Kenya; 5 Département des Eaux et Forêts de l’Ecole Supérieure des Sciences Agronomiques, Université d’Antananarivo, Antananarivo, Madagascar; 6 Transdisciplinarity Laboratory (D-USYS TdLab), Institute for Environmental Decisions, ETH Zurich, Zurich, Switzerland; Shahjalal University of Science and Technology, BANGLADESH

## Abstract

The significant resource investment in research on ecosystems for development of the Global South does not necessarily result in high levels of research knowledge utilisation (RKU). Understanding the factors associated with various levels of RKU can inform funding agencies and researchers developing new projects. We applied a combination of a questionnaire survey and follow up interviews with members of research teams of multiple, broadly comparable projects to make an assessment of achieved RKU levels using a combination of quantitative statistical hypothesis testing and narrative description of survey responses. Research knowledge dissemination by members of the project team who work for non-academic institutions or champions, e.g. particularly motivated people that promote and facilitate implementation or adoption of the project results, and via television was associated with higher research knowledge utilization. By contrast, dissemination by members of the project team working for academic institutions and via peer-reviewed journals was associated with lower RKU. The achieved level of RKU was consistently lower than the targeted level of RKU across spatial scales. The discrepancy between the perceived level of RKU and the evidence provided by survey respondents indicates the need for better monitoring the utilisation of research knowledge in development pathways. Our results further suggest that three years project duration is too short to achieve high levels of RKU in socio-ecological systems. We recommend involvement of non-academic members of the project team in project design, leadership and dissemination for increasing RKU.

## Introduction

Human-induced changes in ecosystems play an important role on global challenges like climate change as well as on the living conditions of local communities [1, 2]. This multi-scale range of potential impacts explains the importance of ecosystems for achieving sustainability transformation [3], as well as the need to address open questions regarding impacts and trade-offs of ecosystem management options on development pathways at local, national, regional or global levels [4, 5]. Therefore, there is a fundamental interest in research on socio-ecological systems as a key element for achieving sustainability pathways.

Conducting research does not guarantee a contribution to sustainable development, however, and it is equally important that non-academic development actors use the insights, knowledge and technologies resulting from research on socio-ecological systems (research knowledge) for policymaking as well as for planning, funding and implementing ecosystem management practices. Following Jacobi et al. [6], research knowledge may include findings of scientific research, including any type of knowledge co-created during research projects, and we are aware that not all co-created knowledge needs to be characterized as “scientific” [7]. Therefore, research knowledge utilisation (RKU) needs to go beyond the realm of the research teams and permeate social actors that are not necessarily involved in the research. Otherwise a gap between science and society arises where access, understandability and appropriateness of science does not match with the requirements of sustainable development pathways [8–10]. Two complementary ways have evolved to avoid or bridge such gaps: evidence-based decision making and transdisciplinary research. While transdisciplinary research proposes an approach where different disciplines as well as representatives of multiple social groups are active in co-producing research knowledge [11, 12], evidence-based decision making focuses on the transmission of research outputs to non-scientists [13–15].

Despite decades of inter- and multidisciplinary research, the gap between scientists and possible users of knowledge outside the projects’ realm persist [14, 16]. Similarly, measuring the impact of transdisciplinary research on development pathways remains challenging [17, 18]. Furthermore, the contribution of research to development pathways in the Global South has been understudied [6, 19]. We propose that understanding who uses research knowledge, for what and which scale (i.e. Research Knowledge Utilization) can be used as proxy for assessing impact of this research on development pathways. In our research we use the analytical framework on stages of utilization of knowledge as presented in [6] that includes understanding who beyond the members of project teams uses research knowledge, for what purpose and at which scale, as well as eliciting enabling and hindering factors of utilization of research knowledge. The starting point in our analytical framework are the stages of knowledge defined for ladder of RKU of [20], who identified six levels of RKU that range from informing stakeholders about research results to practical application of the research knowledge (Table 1).

**Table 1:** Stages of the ladder of knowledge utilization. Adapted from [20].

Transmission	Transmission of research results to stakeholders through reports or presentations
Cognition	Research results seen and understood by stakeholders
Reference	Work cited as a reference in reports, studies, and strategies of action
Effort	Efforts were made to adopt the results by practitioners and professionals
Influence	Results influenced the choice and decision of stakeholders
Application	Results gave rise to applications and extension by practitioners and professionals

Previous research on patterns or common factors in published studies on RKU may result in hypotheses about the underlying mechanisms and can enhance understanding [21], but formal testing of such hypotheses is rarely done [22]. The existing studies often are (narrative) reviews of the literature or comparisons of a small number of case studies. One cause may be the methodology that is commonly used by scientists in the field, which either does not yield data that can be used as input for statistical tests [21, 23] or the use of formal statistical testing is not widespread among the scientists studying research knowledge. Formal statistical testing would reduce potential bias in interpretation of the results and may contribute to a more solid evidence base to support interventions to enhance RKU.

We set out to assess the perception about the level of RKU by conducting a questionnaire survey among project team members of eight ecosystem related projects in the Swiss Programme for Research on Global Issues for Development (r4d programme; http://www.r4d.ch/). This Programme is a joint funding scheme of the Swiss Agency for Development and Cooperation (SDC) and the Swiss National Science Foundation (SNSF) focusing on reducing poverty and global risks, and contributing to global sustainable development. With the objective of securing the relevance of the research towards development priorities in the global South, all projects are designed and implemented by partnerships between organisations in the global North and in the global South. Six projects in the programme deal with questions related to how ecosystems can be used sustainably for reducing poverty and how negative environmental influences can be reduced within the context of sustainable development.

Using the survey results as input, we tested statistically which characteristics of the project team and stakeholder interactions influence the extent of utilisation of knowledge generated by these projects. Our hypotheses related, in general terms, to geographic scale, the frequency and type of communication of project-generated knowledge and to the role of survey respondents had in their respective projects. Specifically, we addressed the following hypotheses:

1. Perceptions of utilisation of research knowledge varies across different users (participants, stakeholders and beneficiaries) and geographic scales.
2. Increased communication of research output to target groups is associated with increased utilisation of research knowledge and application in policy decisions and adoption of new technologies.
3. Projects that adapted their strategy for increasing utilisation during the course of the project achieve higher levels of utilisation of research knowledge.
4. Projects with many pre-existing collaborations/partnerships achieve higher levels of utilisation of research knowledge.
5. Co-creation of knowledge and highly intradisciplinary teams are associated with the highest levels of utilisation of research knowledge.

During follow-up interviews we further explored some of these factors. Besides the thematic similitude, the projects share the programme’s objectives towards scientific research that contributes to sustainability transformation. Furthermore, all eight projects are implemented by North-South-South research partnerships.

## Methods

For the purpose of this study we used the analytical framework developed by Jacobi et al. [6], where usability is enunciated as one of the focal areas in which transdisciplinarity can address societal problems. Usability focuses on the relevance, effectiveness, and accessibility of research knowledge for participants and other user groups; and the different actors reflect on usability throughout the research process. ([24], as cited by [6]). According to this framework understanding the utilisation of (co-created) knowledge can be used as a proxy for assessing the impact pathways from inter- and transdisciplinary research projects [6].

### Data collection

An online survey was carried out at the end of 2019 to asses and quantify the level of RKU in eight projects in the Swiss r4d Programme (www.r4d.ch): four projects in the Ecosystems Module and four in the Thematically Open Module that dealt with ecosystem related topics (Table 2). Six of the projects had a duration of six years and two three years; the three-year projects had finished at the time the survey was administered. One of the six-year projects was in its third year, whereas the other three were in their fourth year. The survey was sent to 150 researchers and non-academic team members of these eight projects. Addresses of all team members, including project leadership, scientists, assistants and students, were obtained from the coordinator of each project. The survey was open for about a month and two reminders were sent to people who had not responded. The survey (S1 File) primarily asked about the targeted and the achieved levels of RKU using the framework of Landry et al. Each respondent was asked to indicate the targeted and achieved levels of RKU at five spatial scales, ranging from local to global. To substantiate the perceived level of research knowledge, respondents were asked to provide evidence of the highest level of RKU they indicated. In order to understand what affected the level of utilisation, questions were asked about the project partnership, interactions with stakeholders (i.e. parties with a stake in the issue, but who are not member of the project team), characteristics of the respondents as well as barriers to RKU and strategies to overcome these barriers. Because several people have been involved in multiple projects, they were asked to respond for a single project and indicate the project their responses refer to. The respondents and interviewees all consented to participating in the study and to the anonymous use of their responses for scientific research purposes, by filling the questionnaire or verbally.

**Table 2:** Short descriptions of the studied projects. Two projects for which no responses were obtained are not listed. More information can be found on www.r4d.ch.

Short name	Topic/aim	Geographic scope (ISO2 country codes)	Duration (years)	r4d Module
AlaReLa	Fostering resilience in the Alaotra social and ecological system, to reconcile the continuously increasing demand for agricultural products while balancing a growing number of values and interests, through delivery of data and information on drivers and barriers of livelihood opportunities and threats to inform policy and decision-makers for the sustainable use and management of the landscape’s natural and agricultural resources	MG	3	Thematically Open
ProBe	Assess the prospects of sustainable biomass energy value chains in rural–urban contexts in East Africa, with a view to contributing to the formulation and implementation of knowledge-based energy policies that improve urban populations’ access to energy for cooking and safeguard smallholders’ income opportunities	KE, TZ	3	Thematically Open
Forest Transition	Increase the understanding of tropical forest transitions in conjunction with prevailing policy and management regimes, in particular land tenure and payments for forest environmental services schemes, and contribute to strengthen rural social-ecological resilience	VN	6	Ecosystems
Telecoupled landscapes	Devising and testing innovative strategies and institutional arrangements for securing ecosystem service flows and human well-being within and between combined socio-economic and environmental interactions between two or more distant socio-ecological systems, i.e. within and between telecoupled landscapes	LA, MG, MM	6	Thematically Open
OPAL	Explore alternative scenarios for oil palm expansion to inform policy and land use development in Indonesia, Colombia and Cameroon. These scenarios, developed with local communities and oil palm companies, will merge the social, economic, and ecological drivers of oil palm development	CM, CO, ID	6	Ecosystems
Woody Weeds	Help mitigate the negative effects of woody invasive alien species on biodiversity, ecosystem services and human well-being in Eastern Africa, by assessing socio-ecological impacts of such species and co-developing and test-implementing management solutions with local communities	ET, KE, TZ	6	Ecosystems

To verify and better interpret the responses, semi-structured interviews were held with selected respondents. Based on preliminary analysis of the survey responses, these interviews were aimed at the interviewees’ understanding of co-creation of knowledge and the selection of partners and stakeholders that the projects interacted with. Interviewees were also asked about recommendations for project sponsors and managers of future projects with respect to improving the utilisation of research knowledge. A total of eleven respondents were interviewed, from five of the projects. Interviewed participants included project coordinators and scientists, and they were based in Switzerland and countries in the Global South.

### Data analysis

The survey yielded 32 responses from six projects (20% response rate). Three respondents were excluded: two because the respondents answered too few of the questions to be used in the statistical analyses and one because the respondent indicated that the responses about the level of RKU were inaccurate. One of the six projects was excluded because only a single response was obtained.

The levels of knowledge utilisation as defined by [20] do not necessarily occur sequentially, especially if some of the knowledge is co-created and members of the project team participate in the knowledge creation. However, for this study we looked at the utilisation of knowledge that happens externally to the project, i.e. once the project communicates its research knowledge to third parties (‘transmission’ in the Landry framework). The responses were converted into a numerical six-point scale with ‘transmission’ coded as the lowest and ‘application’ as the highest level.

The results were analysed with generalised linear models (using the function glmmTMB in the glmmTMB package in R [25]) with the targeted and achieved levels of RKU as response variables and projects as random explanatory variable. We assumed that the data follow a truncated Poisson distribution, because the data did not include zeros. We also analysed the difference between targeted and achieved levels of RKU as response variable, which followed a normal distribution. Fixed explanatory variables included geographic scale, the number of stakeholder interaction types employed by the project, the number of disciplines represented in the project consortium (the latter two were based on ticked boxes in the survey), how much of the project outputs were based on interdisciplinary collaboration, the frequency of stakeholder communication employed by the project, whether the communication strategy was changed during the course of the project, what role the respondent had in the project (ranging from project coordinator to students) and whether the respondent was based in the Global North or the Global South.

Few of the explanatory variables explained a significant amount of variation in the responses and, because we suspected that this may be due to variation in responses given to questions that were answered using tick boxes, additional models were run to assess whether single levels of the explanatory variables were associated with the level of utilisation. These models were similar to the main model, but were run for individual explanatory variables with the factor levels as explanatory variables with binary responses. The most relevant of these factors in the model (in addition to geographic scale) were identified by dredging, i.e. selection of models that explain most variation based on the lowest Akaike Information Criterion value. The significance of the factors included in the three best models (which always included geographic scale as explanatory variable) was then tested using generalised linear mixed models with these factors only and we report only the factors that were confirmed to be significant using this approach. We assessed whether the effect of the different explanatory factors was similar across geographic scales, which was tested statistically by including the interaction term in the analysis. Since the interaction was never significant, which indicates the effects were indeed similar across geographic scales, we simplified the model by omitting the interaction term from the models presented here. We chose this approach as we didn’t have *a priori* expectations about the most effective factors that may be associated with increased levels of RKU. Hence, we interpret the relationships between the identified factors and the level of utilisation as correlations and not as causal relationships.

## Results

### Main analysis

**Fig 1:**
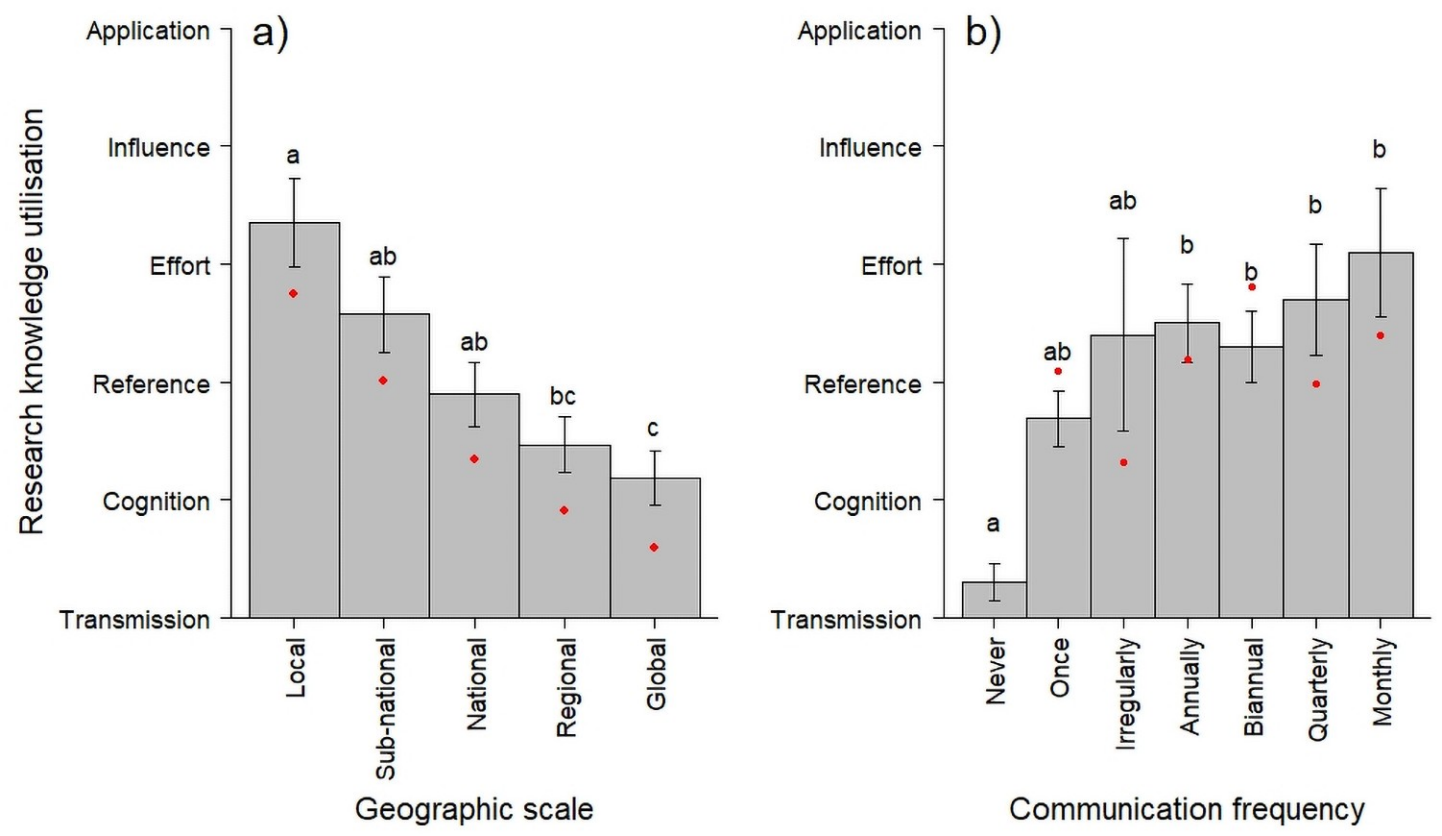
a) The relationship between geographic scale and the extent of achieved RKU and b) the extent of achieved RKU as a function of the frequency of communication of research findings to stakeholders. The level of utilisation is plotted on the scale defined in [20]. Bars indicate the means of the responses and error bars indicate one standard error measure of the mean. The dots indicate the predicted means. The first dot in Fig 1b would be located below the Figure.

A significant negative relationship between the level of RKU and geographic scale was found (hypothesis 1; Table 3; Fig 1a). The average level of utilisation on the global scale was lower (between ‘cognition’ and ‘reference’) than that on the subnational and local scales and the level of utilisation on the local scale (between ‘effort’ and ‘influence’) was significantly higher than on the global, regional and national level. Further, a significant effect of the frequency of communication with stakeholders on the level of RKU was found; regular communication during the project lifetime was associated with significantly higher levels of utilisation (between ‘effort’ and ‘influence’) than when no communication occurred (‘transmission’ only) and single or irregular communication of results were not significantly different from either when no communication occurred or when regular multiple communication had occurred (hypothesis 2; Fig 1b). Finally, the level of achieved RKU was significantly higher in six-year projects than in the two three-year projects and the one project that was in its third year when the survey was administered (below ‘cognition’ and above ‘reference’, respectively). No significant effects of the disciplinary diversity of the project consortium (number of disciplines; hypothesis 5), the pre-existence of collaborations among consortium members (hypothesis 4), changes in strategy during the course of the project to increase RKU (hypothesis 3) or characteristics of the respondents (hypothesis 1) on the level of RKU were found.

**Table 3:** Analysis of deviance table (type II Wald chisquare tests) indicating significance of different factors on the mean level of targeted and achieved RKU (left and right, respectively).

	df	Targeted		Achieved	
		Chisq	P	Chisq	P
Geographic scale	4	28.99	<0.001	30.60	<0.001
Number of interaction types	1	0.32	0.574	1.09	0.296
Project duration	1	0.03	0.867	3.96	0.047
Number of disciplines	1	0.15	0.703	2.29	0.130
Co-creation	5	5.07	0.407	3.18	0.672
Communication frequency	6	14.77	0.022	18.68	0.005
Strategy changed	2	2.34	0.310	2.55	0.279
Role in project	5	3.61	0.607	5.46	0.363
North or South partner	1	0.61	0.435	2.57	0.109

The analysis of the level of research knowledge that the projects aimed to achieve, as indicated by the respondents of the survey, yielded very similar results as the analysis of the achieved level of RKU (Table 3). A clear negative relationship with geographic scale was found that looked similar to, but with consistently higher values than the achieved level of RKU: on average projects aimed for ‘application’ at the local scale and between ‘cognition’ and ‘reference’ on the global scale. The only other factor that explained a significant amount of variation among the responses was the communication frequency; projects that communicated results on a quarterly basis targeted significantly higher levels of RKU than projects that never communicated and all other values were intermediate and not significantly different from either of these frequencies.

On average, the targeted level of RKU was 0.77 higher than the achieved level of RKU. The difference between the targeted and achieved level of RKU was significantly larger in three-year projects than in 6-year projects (0.93 and 0.71, respectively; *P* = 0.009), but the significant effects of two other factors was not as clear. The difference was greater if the communication strategy was changed than when it was unchanged (0.97 vs 0.80; P = 0.006) and although communication frequency had a significant effect on the difference (P < 0.001), no clear pattern emerged.

The evidence for the achieved level of RKU provided by the respondents was diverse. Examples of ‘application’ were cited, as local communities tested or adopted practices developed by the projects, such as the adoption of management practices for woody species that were selected by stakeholders (“Woody Weeds” project), use of mobile phone applications for data collection (“Forest Transitions” project) or implementation of participatory actions to promote sustainable land use (“Telecoupling” project). In many cases, the evidence referred to scientific publications, reports, posts on social media and websites, as well as meetings with or presentations to stakeholders. The latter types are, in the Landry framework, examples of ‘transmission’, although the respondents indicated that higher levels of RKU were achieved. Participants in the three-year projects and the one project that was in its third year emphasised that the impact pathways continue evolving after the research project comes to an end, thus achieving higher stages of RKU over time.

### Factor-level analysis

#### Methods of disseminating results

The analysis of individual factor levels revealed that only a few were significantly associated with differences in the level of RKU across all geographic scales (Table 3). Dissemination of results via television was associated with higher levels of utilisation (close to ‘effort’ instead of close to ‘reference’), but only five respondents of three projects indicated that their projects used this dissemination method. Dissemination via peer-reviewed journal articles was associated with significantly lower levels of RKU, but the average level was only a little lower (7.6% change in mean score) and remained close to ‘reference’. When respondents indicated that non-academic members of the project team were responsible for availing the research knowledge this was associated with higher (between ‘reference’ and ‘effort’), and when scientific members of the project team were responsible for the dissemination with lower levels of RKU (between ‘cognition’ and ‘reference’). These results were due to very few responses, as seven respondents gave similar answers indicating that both groups are responsible; three of the four respondents who didn’t indicate that non-academic members of the project team are responsible for disseminating results work in academic or research institutions (and they did indicate that scientific partners are responsible). There were no significant differences in the level of RKU if the scientific knowledge was availed to stakeholders as peer-reviewed manuscripts or as reviews or non-expert interpretations of the results.

#### Stakeholder interactions and involvement during the project

Several characteristics of stakeholder interactions were considered in the survey, to assess how different ways of interacting with stakeholders affect knowledge utilisation. Overall, co-creation of knowledge was not associated with significant differences in the level of RKU (hypothesis 5; Table 3). There were no differences in the level of RKU between respondents who indicated that this did happen and those who indicated that it did not happen in their project; the significant differences in RKU associated with co-creation during the first or the second half of the project were due to respondents who didn’t know whether co-creation happened in their project. Hence, the survey results indicate that there was a lot of uncertainty about whether co-creation of knowledge occurred in the projects. We therefore decided to explore co-creation as part of the structured interviews.

One of the perceived barriers to successful utilisation of research knowledge was the lack of financial incentives, which was associated with higher levels of utilisation (between ‘reference’ and ‘effort’ instead of between ‘cognition’ and ‘reference’; Table 4). However, respondents who indicated that this was a barrier were not more likely to indicate that financial incentives were paid in their projects. In fact, many of the respondents indicated that people were compensated financially for participating in activities organised by the projects, which may reflect that projects effectively addressed this potential barrier by paying for transport, accommodation and sometimes for lost time (per diems).

**Table 4:** Factor levels significantly affected with differences in RKU on the scale defined by [20].

	No	Yes	P	Percent change
*Stakeholder interaction types*				
Dissemination of results using TV	2.80 (0.16)	3.80 (0.37)	0.006	35.7
Dissemination of results using peer-reviewed papers	3.19 (0.18)	2.95 (0.24)	0.043	-7.6
*Responsible for disseminating results*				
Non-academic partner	2.19 (0.29)	3.54 (0.50)	0.006	61.6
Scientific partner	3.29 (0.44)	2.36 (0.33)	0.049	-28.3
*Barriers*				
Lack of financial incentives	2.37 (0.24)	3.27 (0.41)	0.006	38.0
Research is perceived to be irrelevant or unhelpful	4.04 (0.39)	1.92 (0.32)	<0.001	-52.5
Suggestions are not realistic, relevant or applicable in the local context	2.08 (0.21)	3.72 (0.55)	<0.001	78.8
*Solutions*				
Improved communication at all levels of utilisation	2.29 (0.25)	3.07 (0.20)	0.016	34.1

The steps on the scale were converted into numbers ranging from 1 (‘transmission’) to 6 (‘application’). Indicated are mean values (+/-SE), P values and the percentage change.

Two other potential barriers were significantly associated with differences in the level of utilisation of research knowledge. First, when respondent perceived “research is perceived to be irrelevant or helpful” as a barrier, this was associated with lower utilisation (‘Cognition’). Second, when “suggestions are not realistic, relevant or applicable in the local context” the associated RKU level was between ‘reference’ and ‘effort’. It is possible that the projects anticipated these (potential) barriers and engaged the stakeholders in co-design of the project idea and proposal, hence the research is considered to be relevant, and engaged them in co-creation of knowledge during the project that led to relevant and locally adapted outputs, which then resulted in comparatively high levels of RKU in the projects.

Different types of training did not affect the level of RKU, although many respondents indicated in an open-ended response that student training was a successful strategy to increase the level of RKU. Improved communication at all levels of utilisation was the solution that positively associated with the level of research knowledge (‘reference’). When asked in the survey what would improve RKU, 22 respondents (ca. 71%) indicated that they thought increased communication (e.g. transmission, reference and effort) would increase the level of RKU in their project. The respondents also mentioned that participation of stakeholders in workshops was one action or strategy that worked best to achieve the targeted level of RKU, although they didn’t specify when such workshops were held. Involvement of stakeholders in research activities, for example by collecting data, were not significantly associated with different levels of RKU.

### Interview responses

The interviews were held with eleven survey respondents (over 30% of the respondents), representing five of the six projects included in this study. Five people were based in Switzerland and six in African and Asian countries. The Swiss interviewees were project coordinators, senior scientists and one project administrator and the other interviewees had various roles, including principal investigators in partner countries, PhD students, senior scientists and administrators. Almost all interviewees worked at academic institutions. The interviews were guided by the three main questions below, but also touched on other topics, such as the identification of partners for collaboration in new projects.

*What is co-creation of knowledge and (how) did your project use it*? Some of the respondents were not familiar with the term co-creation, but all did describe creation of knowledge in their project in terms that indicate that at least some of it was the result of interactions with non-academic stakeholders who were not members of the project consortium, such as farmers, local politicians or industry groups. The methods of co-creation of knowledge varied among projects and co-creation happened at different stages of the project; in some cases, stakeholder knowledge influenced the design of project activities during project development or during the initiation phase (co-design). One interviewee indicated that this was difficult because of a lack of time during the project development phase and a lack of funds at the very start of the project.

*What are champions or key contacts and how do you identify them*, *partners for a research project team and stakeholders who are not members of the project team but who participate in project activities*? Many of the collaborations with partners (i.e. members of the project team) were based on existing partnerships and good previous collaborative experiences; some of the interviewees indicated a history of collaboration dating back decades. In other cases, new partners were identified based on recommendations by existing contacts or identified based on academic publication records or professional network that the new partner had. In certain cases, potential collaborators were identified as institutions and then the most appropriate participants from those institutions were identified. Finally, one interviewee expressed the opinion that collaborations are based on shared interest and that one key attraction of collaborating in international projects is the promise of receiving funds when people in the Global South participate as project team members.

Stakeholders who were not part of the project team were often identified by existing local contacts or institutions and the mechanism appeared to be specific to the local context; in some cases, recommendations were made by existing contacts and in other cases a selection was made by local institutions or administrations after an explanation of the project aims and activities was provided. One interviewee mentioned that involvement of additional stakeholders was based on recommendations made by stakeholders who participated in project activities. Interviewees of at least two projects mentioned that a stakeholder analysis or mapping was carried out to identify relevant stakeholders or stakeholder groups.

Different opinions were expressed concerning the best, i.e. most influential individuals to involve, either as member of the project team or as stakeholders that are not part of the project team. What is best appears due to local and institutional settings: some interviewees indicated that politicians are most influential, whereas other interviewees said the opposite and career civil servants were believed to have more and longer lasting influence. In two cases, interviewees mentioned the cultural history of the countries that they work in that influenced the selection of stakeholders, which appeared specifically related to formerly communist countries. While the importance of champions, e.g. particularly motivated people that promote and facilitate implementation or adoption of the project results, was highlighted in the survey, several interviewees said that it is impossible to predict which individuals will become champions. Some interviewees also indicated that successful stakeholder interaction, and even collaboration with new members of the project team, rely on an element of chance. Examples of encounters with politicians or exceptionally motivated or influential stakeholders that led to significant advancement of RKU were cited.

*What would you recommend donors and people developing new projects with respect to the project design and who to involve*, *how and when*. Several interviewees indicated that a thorough understanding of the local context will lead to more relevant research. Multiple interviewees suggested that understanding of the context and communication may be improved if some of the Swiss participants have a basic understanding of the local language, have worked in the country prior to submission of the project proposal, or spend significant time in the country during project implementation (for example students doing field work, or reconnaissance missions prior to submission of project proposals). Good knowledge of English was highlighted by a few interviewees as an important facilitator for good collaboration. It was recommended by several interviewees that all members of the project team should be involved early in the project development. They should have significant input in the co-design of the project to ensure that the proposal reflects input and interest of all proposal consortium partners. These actions would require funding before the project is secured and some interviewees indicated that donors should be prepared to provide this funding even if the risk exists that not all project proposals would be accepted. Other interviewees indicated that such resources can be made available as investment into potential future projects by some partners. Several interviewees also suggested that more budgetary flexibility should be given by the donor so that projects would be able to adapt their activities, if stakeholder interactions at the start of the project indicate that some adjustments would result in more relevant or otherwise appropriate outputs. Several people indicated that projects need resources to develop and maintain strong relationships among consortium partners, in particular in situations where some did not collaborate previously, as well as relationships with stakeholders. Two interviewees highlighted the importance of fostering good personal relationships among consortium partners by reserving enough time for socialising during meetings and the need, especially for project leadership, to be openminded about other viewpoints and willingness to adapt your own opinion. One interviewee mentioned the value of diplomatic skills to allow participants to change their opinion (without losing face). This seems particularly relevant in inter- and transdisciplinary projects in view of the often-perceived gap between scientists and non-academic members of the project team that may hamper successful communication of research findings to stakeholders.

## Discussion

### Factors associated with high or low levels of RKU

The level of utilisation of knowledge generated through research for development projects is often disappointing and, while the factors associated with the level of RKU have been studied in different fields, few studies have looked at what promotes RKU in research for development. Our analysis reveals a number of factors influencing the level of utilisation of knowledge produced in the context of research for development in the socio-ecological systems. We found a higher level of RKU at local than at the national and global levels and six-year projects had a higher level of RKU than three-year projects. The clear relationship between geographic scale and the level of RKU may be expected, as the survey respondents indicated that their projects targeted and thus were likely to achieve more on the local scale than at the national or global level, especially during the short time frame of these projects. Dissemination by non-academic members of the project teams and the barriers related to the relevance of research and suggestions by the projects explained the largest percent changes in scores, indicating the importance of these factors when considering what promotes RKU. These results are broadly similar to those reported for other sectors, such as education [26] or social work [23], and provide indications for best practices to overcome the gap between researchers and practitioners.

The analysis of factors that enhance RKU beyond the sphere of the projects points towards the important role of non-academic members of project teams as early as project development or the start of implementation, through involvement of relevant stakeholders, as dissemination done by them and/or champions is most effective for achieving higher RKU levels. In addition, although the publication of results in scientific journals is an important way to secure stages like transmission, cognition or reference, the use of other, non-scientific communication media (i.e. television) was related to higher levels of RKU. Television was one of the potential means of communication of results that respondents could select, but it was the only of these communication tools that was found to be significantly associated with higher levels of RKU. Aligned with this result is the involvement of non-academic partners in project co-design and knowledge co-production, that arose as factors for getting higher RKU in the interviews. Finally, the achieved level of utilisation was consistently lower than what was aimed for, but the reasons explaining this shortcoming were not fully explored during our study. We recognise that, as the majority of the projects included in the study were still ongoing, a further increment of RKU can be achieved before and even after finalisation of the projects. Additionally, it is necessary to recognise that the design of projects depend upon applicants as much as upon the expectations and modalities from funding agencies, and it may also be that project proposals are ambitiously worded with the aim of securing funding [27, 28].

A number of barriers to RKU were identified, including the lack of financial incentives and the appropriateness of the produced knowledge. However, it is unclear how to interpret some of them, as the survey responses seem contradictory. For example, many of the respondents who identified the lack of financial incentives as a barrier to RKU also indicated that people were compensated financially for participating in activities organised by the projects, by paying for transport, accommodation and sometimes for lost time (per diems). Thus, it appears that this perceived barrier may be potential only and that projects responded to this challenge through their actions and strategies. Similarly, almost all interviewees indicated that co-creation of knowledge was used and that stakeholders should be involved from the initiation or inception meeting of the project, leading to co-design of research that addresses relevant issues and yields suggestions that are adapted to the local conditions. Hence, our results suggest that recognition of (potential) barriers to RKU may have led to the implementation of mitigation measures by the projects.

### Strength and criticism of the methodology

While our results are largely in agreement with knowledge about RKU in other domains, they are based on a small number of responses and projects and must be interpreted with caution. The examples of Newig et al. [22] and de Jong et al. [29] show that such analyses can be done on large datasets, but in order to keep a certain level of comparability our sample was limited to the research projects dealing with ecosystems within the Swiss Programme for Research on Global Issues for Development (r4d Programme) through the system boundaries given by the r4d programme. Expanding our analysis to a larger number of projects with similar thematic was impossible within the Swiss r4d Programme. In addition, we didn’t try to establish causality, but rather tried to show relationships, and we highlight below how important is to monitor RKU in the future in order to better understand impact. Still, a larger sample would enable verification of our findings and possibly allow to draw more general conclusions; it would therefore be useful to perform a quantitative analysis study of RKU among all funded r4d projects. Our study revealed the practical advantage of adopting an incremental scale: the scores were converted into numerical values and these were analysed using statistical regression models [22, 29]. However, while a larger sample size would strengthen the statistical power of the analysis, the data would probably be more variable as a result of the greater geographic and topical amplitude of the projects.

A strength of our study is that we collected multiple responses per project, and it thus considers the diversity of project team members (in terms of location, discipline, role, etc.). The diversity is reflected in the variation among responses, indicating that the responses depend on differences in perceptions or knowledge among members of the project teams. This is an improvement over the narrative reviews that are a common way of comparing studies of RKU and the two larger studies of Newig et al. [22] and de Jong et al. [29]. Newig et al. [22] obtained single responses for each of 81 projects by asking project heads to fill a questionnaire. De Jong et al. [29] obtained multiple responses for some of the studied projects (440 partial responses for 178 projects), and if one response was received of a project it was preferably provided by the project leader. That some project characteristics in our study explained a significant amount of variation in the data despite the variability of the individual responses obtained from the project participants appears to confirm the robustness of the results. Discussion of specific circumstances, both promoting and hindering RKU, is particularly valuable to explore why differences or variation in the level of RKU occur and we aimed to do this through the interviews. The combination of the survey and follow-up interviews in our study revealed both generalities and specific externalities that were specific for individual projects.

In this study, the model of Landry et al. [20] was adopted, but other models that describe RKU exist, and the difference appears mainly to result from the way researchers and users of knowledge interact during knowledge creation. For example, the social or interaction model puts emphasis on co-creation of knowledge with stakeholders who use the knowledge [30]. In this study, we decided to specifically assess RKU by stakeholders beyond the research consortium and we thus focus primarily on use rather than generation of research knowledge. While co-creation is probably a common part in research-based (development) work and implementing stakeholders are part in this process [6, 23], the importance of co-creation of knowledge may depend on the geographic scale and the stage of the project.

### Recommendations: Better recording of knowledge use

The overestimation of research use, revealed by the proffered evidence for lower levels of RKU than indicated, is similar to reported in the review by Adams et al. [31] and our results indicate that a better monitoring of progress in the utilisation of research knowledge during and after research activities is needed. Many funding agencies have requirements with respect to reporting of evidence for the achievement of defined levels of RKU, which may be used for project evaluation. The Swiss r4d Programme that funded the projects in our study doesn’t have specific requirements with respect to reporting on the achieved RKU and does not use such evidence for assessing project success. The results of our study show that the measures of success the respondents cited are insufficient to show the extent of RKU that is achieved and that suggests that some respondents don’t know well what happens with the research knowledge beyond transmission or reference. Specifically, many of the examples of evidence for RKU do not reflect whether anything happened with the knowledge beyond transmission. We expected that at least the project leadership would be aware of how project outputs are being used, but they also didn’t necessarily provide responses that reflect that. The suspected lack of awareness was not confirmed during the interviews, where people explained in some detail the utilisation of their knowledge. Hence, it does not appear that there are political reasons for the overestimation [27] or that the misrepresentation is strategic [28].

While it is unlikely that change, or application of knowledge will be achieved during the lifetime of projects, it is possible to better document intermediate levels of RKU, including reference, effort and influence. Hence, it would be relevant for projects to record how knowledge generated is being utilised beyond the transmission stage, and projects should document and report intermediate levels of RKU at least by recording page views, links, likes and retweets, as well as citations. This appears especially pertinent in light of the interviews, where people indicated that such records are important for identification of collaborators for future projects and to show donors and reviewers of project proposals the impact the collaborators have had in the past. Some interviewees indicated that if the project had more funds, would it have been possible to use the time of developing and implementing strategy to collect and evaluate data on RKU and a more generous resource allocation for monitoring and evaluation would allow this to happen in future projects.

Partnerships are well established in the r4d arena, as illustrated by the r4d programme, and the frequent reference to co-design and co-creation during the interviews, as well as the evidence from the survey, which shows the higher level of RKU when dissemination was done by non-academic partners. We interpreted the model of Landry et al. [20] as a linear model to characterise RKU outside of the project consortium, whereas some of the respondents prefer an interaction model because they believe the separation between producers and users of knowledge in the sustainable development context is not clear. The Landry model may be particularly valuable in situations where knowledge generated in r4d projects is upscaled or outscaled without the same level of interaction between scientists and stakeholders. In these cases, the identity of producers and users of the knowledge is easier to define. Newig et al. [22] use a similar scale, a weighted index that ranged from mere recognition to continual implementation of research results by practitioners. Self-assessment of research impact on a similar scale was earlier used by de Jong et al. [29] in a study of transdisciplinary research in a climate science programme in The Netherlands. Recently, users of the Landry model have recognised that multiple stages can happen in parallel within the length of a research project [6]. We acknowledge the possibility of simultaneous stages, but highlight the importance of transmission and cognition as basis for the other stages. In other words, a user of research knowledge beyond the project boundaries needs to get access to this knowledge (transmission) in a way that empowers him/her to achieve further utilisation of research knowledge.

### Recommendations: Project leadership in research for development—Scientists or implementers?

One of the striking, consistent patterns in the results of our study is that communication by scientists and using scientific publications had significant negative impacts on the level of RKU, whereas communication by non-academic partners had a stronger, opposite effect. This confirms the findings by Newig et al. [22]. While this may appear intuitive, it is an important result, because these projects were all coordinated by scientists despite the clear interest of the donor in practical application of the results, and the tension between this interest in translation of research into practical applications and the need for scientists to publish peer-reviewed papers to advance their careers and reputation. It is therefore necessary that r4d partnerships include partners that have outstanding skills in communicating with stakeholders. In the interviews, it was said multiple times that new members of project teams were identified based on their academic record, hence presumably not for communication skills. Further, the academic project leader is rewarded based on peer-reviewed publications and not for the achieved RKU.

Altogether, our findings highlight the need to rethink the role of non-academic members of project teams in the design and governance of r4d projects. This finding is aligned with recent research that highlights the importance of including non-academic actors in research activities related to ecosystems in order to increase not only utilisation, but also relevance and legitimacy of research for development activities [32–34]. This relates to funding mechanisms and (co-)leading organisations, as well as the ultimate focus and purpose of r4d. Actors in this field should consider whether non-scientific organisations can lead successful r4d projects and it also highlights the need for promoting balanced and effective interdisciplinary and transdisciplinary collaboration in r4d project teams. The latter also requires effective communication within the project team. Hence, project sponsors and applicants for funding should consider alternative possibilities for project leadership when considering funding for, and the design of research projects that aim to result in high levels of RKU. Potential mechanisms to promote RKU include entertaining joint applications by research partners and implementing institutions and requiring evidence for the achievement of defined levels of RKU as one criterion for evaluating project success. A reward system based on bonus payments if such criteria are met may provide incentive for projects. The effects of such innovations should be monitored to allow informed decisions to make further improvements.

## Supporting information

S1 File The questionnaire survey used in this study. (DOCX)
