# Supplementary material for: Factors enhancing the level of utilisation of research knowledge on ecosystems
Source: PLoS One. 2021 Jul 22;16(7):e0254752. doi: 10.1371/journal.pone.0254752 (PMC8297782; doi:10.1371/journal.pone.0254752)
Supplement: S1 File — (DOCX) [file pone.0254752.s001.docx]

S1 File, belonging to Eschen et al., “Factors enhancing the level of utilisation of research knowledge on ecosystems”. The questionnaire used in the study.

Successful utilisation of research knowledge in the r4d Ecosystems and Thematically Open Modules

What is it about: The r4d Programme wants to learn about how knowledge, as a key product of research activities, is used in transformative pathways. The Programme has funded several synthesis projects and this one aims at learning from projects in the Ecosystems and Thematically Open modules about successful ways of overcoming barriers to utilisation of research knowledge. With this questionnaire, we aim to obtain responses from several people in each project, to capture the variety of experiences and opinions. We consider a research project as a knowledge production process and the utilization of the knowledge produced a proxy for understanding impact on third parties (farmers, professionals, scientists, policy makers, organizations, processes, etc.). We are aware that the impact of research activities depends upon the utilization of new knowledge by stakeholders at all levels, from local to global, and that your project may not have ambitions on all spatial scales and that you may not (yet) achieve or aim to achieve influence or application on all spatial scales.

The survey will provide information characterizing the utilization of the knowledge you have produced by the multiple stakeholders within and outside your project, according to the stages of knowledge utilization as proposed by Landry et al (2001). This are the six stages of knowledge utilization:

1. TRANSMISSION: The project team transmitted its research results to third parties concerned.
2. COGNITION: the outputs of the project were read /seen and understood by the third parties concerned.
3. REFERENCE: The outputs of the projects have been cited as a reference in the reports, study plans, strategies or policies elaborated by third parties concerned.
4. EFFORT: Efforts were made to (facilitate/secure) adopt the project's results by third parties.
5. INFLUENCE: The projects outputs and results influenced the choice and decisions of third parties.
6. APPLICATION The outputs and results of the project gave rise to applications (e.g. concrete practices at the field) and extension/capacity building activities by third parties.

What we aim to do: We would like to assess your knowledge and perceptions about the utilization of knowledge in your R4D project, but specifically look for evidence for the most advanced level of knowledge utilization to validate individual responses. We are aware that achieving especially the effort and influence stages may be difficult to show objectively, but responses to these questions are highly valuable. Evidence can be highly diverse, including scientific publications, newspaper articles, video, Twitter, Facebook, reports or other. We have a particular interest in barriers to achieving a higher level of research utilisation. In many cases projects will aim to achieve changes in people’s behaviour or implement a new technique or tool, but often this will not be successful (yet). A central question therefore is why the project achieved the level or knowledge utilization that you indicate and whether special efforts were made to achieve this level.

Landry et al. (2001) Climbing the ladder of research utilisation", Sci. Comm., Vol.22, No 4, pp 396-422.

Section 1 – General information about you (responses will be treated anonymously)

1. Name
2. R4D project name
3. Role in project (give options with tick boxes and open-ended?)
4. Discipline (give options with tick boxes and open-ended?)
5. Country based in (name)
6. Countries active (names)
7. Employer type (give options with tick boxes and open-ended?)
8. Gender (M/F/prefer not say)
9. We are looking to interview a few people per project in early 2020 to clarify some of the responses given in the questionnaire. Would you be available for an interview? In return for an interview we offer co-authorship on the written outputs of our project (yes/no)
10. Contacts details (e-mail, phone, skype)

Section 2 – About your project

1. Aim of project (need more specific questions, but I think it would be good to ask this to be able to compare with the responses below)
2. Which disciplines are involved in your project?
   1. What is the ratio scientific/non-scientific partners in your project?
3. How much of the research output of your project is interdisciplinary and how much transdisciplinary?
4. When was co-creating of knowledge done in your project, how much of the co-creation is the result of interdisciplinary collaboration?
5. Stated objectives (could also be taken from website or project proposal, but this may be more appropriate to use as validation)
   1. Targeted geographic scale (local, sub-national, national, regional, global)
   2. What is the targeted level of knowledge utilisation in your project? Provide ticks in the table below:

|  | Global | Regional | National | Sub-national | Local |
| --- | --- | --- | --- | --- | --- |
| Transmission |  |  |  |  |  |
| Cognition |  |  |  |  |  |
| Reference |  |  |  |  |  |
| Effort |  |  |  |  |  |
| Influence |  |  |  |  |  |
| Application |  |  |  |  |  |

- 1. What research knowledge does the project aim to avail, at which geographic scales and to what stakeholders? (open-ended) Did it achieve that? (yes/no/partially). Please specify
  2. What specific actions does the project undertake to achieve the six levels of knowledge utilisation?

1. Who is responsible for availing the research knowledge in the project?
2. Does the project aim to influence policy?
   1. If it does but didn’t achieve it yet, what would be needed to increase project knowledge utilisation in policy formulation?
3. What is the highest achieved level or knowledge utilisation at each of these geographic scales? Provide ticks in the table below:
   1. For each spatial scale, please provide evidence of the level of utilisation being achieved. This can be links, citations, reports, meeting invitations, etc.

|  | Global | Regional | National | Sub-national | Local | Evidence |
| --- | --- | --- | --- | --- | --- | --- |
| Transmission |  |  |  |  |  |  |
| Cognition |  |  |  |  |  |  |
| Reference |  |  |  |  |  |  |
| Effort |  |  |  |  |  |  |
| Influence |  |  |  |  |  |  |
| Application |  |  |  |  |  |  |

Section 3 – Strategies for successful knowledge utilisation

1. Which specific stakeholders does the project aim to interact with (open ended and tick boxes for types and geographic scale?);
2. How did the project identify the relevant stakeholders?
3. How many stakeholders did you target and how many of those were existing contacts for (some of) the project participants?
4. When were the identified stakeholders first contacted for this project?
5. How often have these stakeholders been provided with research knowledge generated by this project?
6. For how many years had you or your organisation been collaborating with the partners in the r4d project? Please name the partners and tick the relevant boxes:

| Partner name | 1-2 years | 3-5 years | 5-10 years | >10 years |
| --- | --- | --- | --- | --- |
| … |  |  |  |  |
| … |  |  |  |  |
| … |  |  |  |  |
| … |  |  |  |  |

1. What kind of interaction was established (face-to-face, partnership, social media (which ones), tv, radio, newspapers, workshops, peer-reviewed publications (international/ English or other), …; tick-boxes and open-ended, for each of the identified stakeholders)
2. If you provided results of scientific research, did you
   1. Provide non-expert interpretations of your own results?
   2. Provide reviews or summaries of the scientific literature on the subject?
3. Are the stakeholders involved in carrying out the research, by providing support, collecting data or other?
4. Does the project train or educate people (no, yes, via training workshops, vocational training, internships, academic on MSc/PhD level, other)?
5. The interactions with which of these stakeholders led to the highest level of knowledge utilisation?
6. According to you, which actions or strategies undertaken by the project worked best to achieve their intended knowledge utilisation targets? (open-ended, for each geographic scale)
7. Has your project provided financial incentives to stakeholders to motivate them to participate in meetings to avail research knowledge or trial or implement research knowledge or tools? (yes/no) Please specificty

Section 4 - Barriers to Knowledge utilisation

1. What barriers were experienced or perceived that may hinder knowledge utilisation?
   1. Limited access to literature
   2. Lack of time
   3. Lack of trust in the findings
   4. Failure to understand the language or the statistics
   5. Lack of financial incentives
   6. Reliance on other sources of information
   7. Research is perceived to be irrelevant, unhelpful and too theoretical.
   8. Lack of motivation
   9. Results/suggestions are not realistic, relevant or applicable in the local context
   10. Other …
2. What solutions were employed to overcome the specific challenges?
   1. Improved communication at all levels of utilisation
   2. Need to be involved in research
   3. Good links between researchers and practitioners
   4. Targeted involvement of affected stakeholders in decisions and research design
   5. Identification of champions
   6. Demonstration trials
   7. Workshops
   8. Public information meetings
   9. More stakeholder interaction at higher level than the target (top-down)
   10. Other …
3. Which specific stakeholders would the project need to interact with, but has not successfully achieved so yet? How could this be achieved?
   1. Please provide the name and contact details of one such stakeholder. We would like to interview one such stakeholder per project, so please provide detailed information.
4. Has the strategy of the project for ensuring knowledge utilisation, or working towards impacts, changed over the course of the project (for example when an extension was sought after three years) and in what way? Has this had a significant impact on the achieved level of knowledge utilisation?
5. In your opinion, would more communication (e.g. transmission, reference and effort) increase the level of knowledge utilisation in the project?
6. Do barriers vary across the knowledge utilisation levels, geographic scales and countries?

|  | Main barrier | Countries affected | Successful and unsuccessful solution | Evidence |
| --- | --- | --- | --- | --- |
| Global |  |  |  |  |
| Regional |  |  |  |  |
| National |  |  |  |  |
| Sub-national |  |  |  |  |
| Local |  |  |  |  |

1. According to you, which strategies undertaken by the project did not yield any results or were less useful in the process? (open-ended, for each geographic scale)
